# A Novel Radiomic Model for Risk Stratification of Cerebral Herniation in Radiation-Induced Cystic Brain Necrosis

**DOI:** 10.3390/cancers18060953

**Published:** 2026-03-14

**Authors:** Hongbiao Hou, Jinhua Cai, Mingyi Bao, Zongwei Yue, Mingwei Xie, Zhaoxi Cai, Yanting Chen, Zecong Lin, Le Zeng, Yi Li, Honghong Li, Yongteng Xu, Yamei Tang

**Affiliations:** 1 Department of Neurology, Sun Yat-Sen Memorial Hospital, Sun Yat-Sen University, Guangzhou 510120, China; houhb5@mail2.sysu.edu.cn (H.H.); caijh56@mail.sysu.edu.cn (J.C.); baomy@mail2.sysu.edu.cn (M.B.); yuezw@mail.sysu.edu.cn (Z.Y.); chenyt367@mail.sysu.edu.cn (Y.C.); linzc3@mail2.sysu.edu.cn (Z.L.); liyi59@mail.sysu.edu.cn (Y.L.); lihh37@mail.sysu.edu.cn (H.L.); 2 Department of Radiology, Sun Yat-Sen Memorial Hospital, Sun Yat-Sen University, Guangzhou 510120, China; xiemw3@mail.sysu.edu.cn (M.X.); caizhx3@mail.sysu.edu.cn (Z.C.); 3 Department of Anesthesiology, Sun Yat-Sen Memorial Hospital, Sun Yat-Sen University, Guangzhou 510120, China; zengle7@mail2.sysu.edu.cn; 4 Guangdong Provincial Key Laboratory of Malignant Tumor Epigenetics and Gene Regulation, Sun Yat-Sen Memorial Hospital, Sun Yat-Sen University, Guangzhou 510120, China; 5 Guangdong Province Key Laboratory of Brain Function and Disease, Zhongshan School of Medicine, Sun Yat-Sen University, Guangzhou 510080, China

**Keywords:** cystic brain necrosis, radiomics, cerebral herniation, radiotherapy, nasopharyngeal carcinoma, magnetic resonance imaging

## Abstract

**Background:** Radiation-induced cystic brain necrosis (RCN) can progress rapidly to life-threatening cerebral herniation. This study aimed to develop a predictive model integrating radiomic features and clinical variables to assess the risk of cerebral herniation in RCN patients. **Methods:** A total of 130 patients diagnosed with RCN following radiotherapy for nasopharyngeal carcinoma were retrospectively enrolled and randomly assigned to training (*n* = 91) and testing (*n* = 39) cohorts in a 7:3 ratio. Radiomic features were extracted from baseline T2-weighted magnetic resonance imaging (MRI), and a radiomic signature was constructed using least absolute shrinkage and selection operator regression. A multivariate Cox regression model was then developed by incorporating the radiomic signature and clinical variables to predict cerebral herniation. The model’s discriminative ability, calibration, and clinical utility were evaluated. **Results:** The radiomic signature based on five selected radiomic features demonstrated good predictive performance. The radiomic model, which integrated the radiomic signature and ratios of perilesional enhancement, exhibited favorable performance in both the training cohort (C-index: 0.841) and testing cohort (C-index: 0.867). The model successfully stratified patients into high- and low-risk groups. The calibration curves showed good agreement and the decision curve confirmed the clinical utility of the model. **Conclusions:** The MRI-based radiomic model, which integrates radiomic features and clinical variables, demonstrates robust performance in predicting cerebral herniation in RCN patients, offering a practical and user-friendly tool to support clinical decision-making.

## Simple Summary

Radiation-induced cystic brain necrosis (RCN) can progress rapidly to life-threatening cerebral herniation. In this study, we identified a radiomic signature derived from baseline magnetic resonance images (MRIs) to stratify the risk of cerebral herniation in nasopharyngeal carcinoma survivors with RCN. By incorporating the radiomic signature and ratios of perilesional enhancement, a radiomic model was developed and showed favorable performance in the training and testing cohorts. Our findings demonstrate that radiomic features extracted from MRI can predict the risk of cerebral herniation in patients with RCN. The radiomic model can serve as an easy-to-use and non-invasive tool for managing patients with RCN. Specifically, patients identified as high-risk should receive more frequent imaging surveillance and clinical monitoring, with surgical intervention considered when necessary.

## 1. Introduction

Radiation-induced brain injury (RIBI) is a devastating and often irreversible complication of radiotherapy for nasopharyngeal carcinoma and can be life-threatening in its most severe forms [1,2,3]. The manifestation of RIBI on imaging corresponds to different pathological stages. During the edematous and necrotic phases, cranial magnetic resonance imaging (MRI) primarily reveals white matter lesions and contrast-enhanced lesions [4]. As the lesions progress to the cystic phase, a subset of patients may develop a distinct subtype known as radiation-induced cystic brain necrosis (RCN). This condition is characterized by cyst-like lesions that appear hypointense on T1-weighted imaging (T1WI), hyperintense on T2-weighted imaging (T2WI), and exhibit a clearly defined cyst wall [5].

As the most severe form of RIBI, RCN has been reported to occur in approximately 1.7–12.7% of cases [6,7]. The pathogenesis of RCN is associated with post-radiotherapy tissue necrosis and liquefaction [8,9], microhemorrhages [10] and hemorrhage [11], as well as disruption of the blood–brain barrier [9,12]. During follow-up, RCN lesions typically demonstrate a tendency to enlarge rather than regress [6]. While some lesions progress slowly or remain stable with minimal impact on quality of life or survival, others undergo rapid expansion. This could lead to a sudden rise in intracranial pressure, manifesting as severe headache and a decline in consciousness, which are ominous signs of impending herniation [13]. Furthermore, no available interventions to date have demonstrated efficacy in reversing the disease process [5,13,14]. Consequently, the development of a model to stratify the risk of cerebral herniation in nasopharyngeal carcinoma patients is critical and of great clinical importance.

Radiomics is an emerging imaging analysis technique that applies data-mining algorithms to extract high-throughput, sub-visual quantitative features from conventional medical images, thereby enabling the construction of models for assessment, prediction, and the reflection of pathophysiological changes in challenging clinical scenarios [15,16,17,18]. Despite its potential, no studies have yet explored radiomic applications for predicting cerebral herniation in patients with RCN. Based on this gap, we hypothesized that radiomics-derived morphological characteristics could serve as biomarkers associated with lesion progression. Therefore, this study aimed to develop and validate an integrated radiomic model combining baseline MRI features and clinical variables to stratify the risk of cerebral herniation in nasopharyngeal carcinoma survivors with RCN.

## 2. Materials and Methods

### 2.1. Study Design and Participants

Ethics approval was granted by the local institutional review board for this retrospective analysis. This study was conducted in accordance with the Declaration of Helsinki. We retrospectively screened all RIBI patients admitted to our center between January 2005 and September 2023. The baseline was defined as the first time detecting a well-formed RCN based on cranial MRI. The demographic, clinical, biochemical and imaging data was collected at baseline (detailed imaging features of RCN are provided in Table S1). Detailed descriptions about inclusion criteria, exclusion criteria, and variables collected at baseline are provided in Methods S1 and S2. Finally, a total of 130 patients with RCN after radiotherapy for nasopharyngeal carcinoma met the inclusion and exclusion criteria. The patient recruitment flowchart is shown in Figure S1.

### 2.2. MRI Appearances of Cystic Brain Necrosis

The diagnosis of RCN is mainly based on opinions from both neurologists and radiologists. A well-formed cystic brain necrosis typically appears on cranial MRI as: (1) a round or oval well-defined lesion exhibiting markedly hyperintense signal on T2WI, (2) a hypointense signal on T1WI, (3) a mainly hypointense signal on T2-weighted FLAIR sequences, (4) a thin or imperceptible wall that demonstrates slight contrast enhancement, (5) surrounded by edema lesion [5,19,20]. A typical MRI appearance of RCN is shown in Figure S2.

In our study, a well-formed RCN lesion was required to have a clearly defined boundary and a volume greater than 1 cm^3^, which ensured reliable delineation of the region of interest for radiomic analysis. For patients with bilateral RCN lesions, the larger and better-defined lesion was selected for delineation.

### 2.3. Endpoint

All patients were followed up with cranial MRI at three-month intervals, and an urgent imaging was performed when clinical information or neurologic symptoms indicated possible cerebral herniation. The endpoint was cerebral herniation secondary to RCN, which is detailed described in Methods S3 and Figure S3. Patients without MRI evidence of herniation were censored at the date of their last follow-up.

### 2.4. Ratio of Perilesional Enhancement

The perilesional enhancement of RCN lesions comprised both ring-like enhancement along the cyst wall and adjacent contrast-enhanced parenchymal components, such as regions of radiation-induced brain necrosis [4,5]. On contrast-enhanced T1-weighted coronal images, the perilesional enhancement volumes were segmented and quantified using 3D Slicer software (version 5.6.2, http://www.slicer.org (accessed on 13 January 2026)). Accounting for the heterogeneity in cyst size, the ratio of perilesional enhancement (RPE) was calculated as the proportion of perilesional enhancement volume to cystic lesion volume, which reflected the relative extent of enhancement. For clinical applicability, we categorized RPE into two categories: extensive enhancement and non-extensive enhancement, using the optimal cutoff value of 28.5% generated by X-tile software (version 3.6.1, https://medicine.yale.edu/lab/rimm/research/software/ (accessed on 13 January 2026). Figure S4 illustrates representative MRI of patients with different RPE.

### 2.5. Workflow and Statistical Analysis

**Figure 1:**
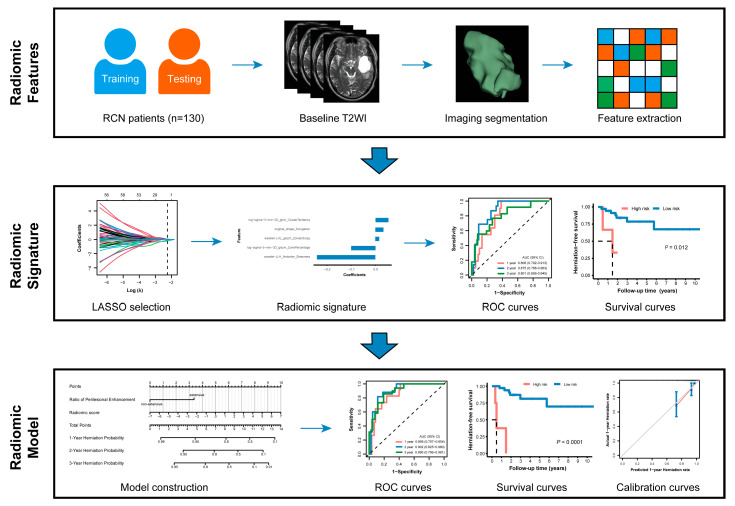
The study workflow.

The detailed information of workflow is described in Figure 1 and Methods S4.

The optimum cutoffs for RPE and risk stratifications were selected using X-tile software version 3.6.1 (Yale University School of Medicine, New Haven, CT, USA) [21]. All statistical tests were performed using R statistical software (version 4.4.1; R Foundation for Statistical Computing, Vienna, Austria). Details of packages used for data analysis are provided in Table S2. A two-sided *p* < 0.05 was considered statistically significant.

## 3. Results

### 3.1. Patient Characteristics

A total of 130 patients (99 males and 31 females) with a mean age of 51.9 ± 9 years were enrolled in this study. The cohort was randomly divided into a training set (70%, 91/130) for model development and a testing set (30%, 39/130) for internal validation. The median interval from radiotherapy to the initial diagnosis of RCN was 9.2 years (IQR: 7.5–12.2). Over a median follow-up period of 2.1 years (IQR: 0.9–4.0), cerebral herniation occurred in 23.1% of RCN lesions. The baseline clinical characteristics of the entire cohort are summarized in Table 1. No statistically significant differences were observed between the training and testing cohorts across these variables (all *p*-values ranging from 0.063 to 1.000).

### 3.2. Radiomic Signature Construction and Performance Assessment

**Figure 2:**
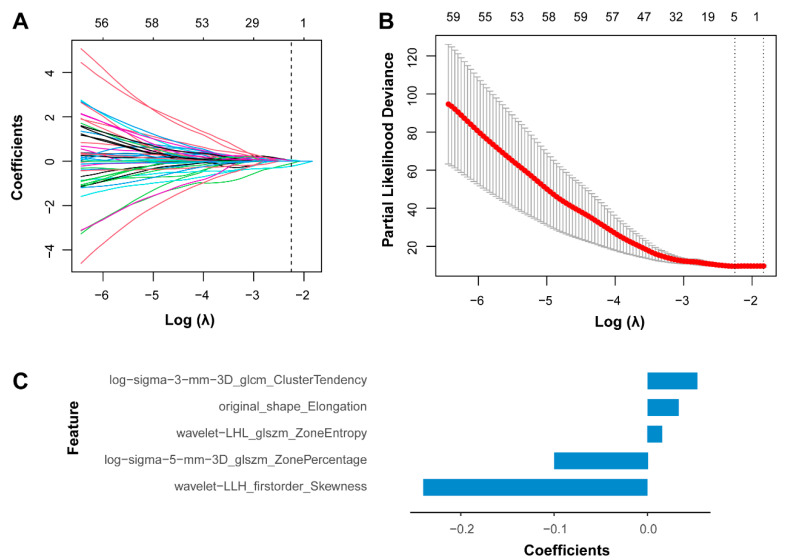
LASSO coefficients of radiomic features. (**A**) The LASSO coefficient profiles of the 1037 radiomic features. A vertical line was generated at the log(λ) value by using ten-fold cross-validation, where the optimal λ value selected 5 radiomic features. The optimal λ value of 0.105 was chosen. The numbers along the upper *x*-axis represent the number of nonzero coefficient features in the model. (**B**) The black, dotted vertical line was drawn at the value selected using ten-fold cross-validation in (**A**). The upper *X*-axis represents the number of nonzero coefficient features in the model. The red dots indicate the average deviance values for each model with a given λ, and the vertical bars through the red dots show the upper and lower values of the deviances. (**C**) A histogram showing the coefficients of the selected radiomic features in the radiomic signature. The radiomic score calculation formula: Radiomic score = 5.352 × 10^−2^ × log-sigma-3 mm-3D_glcm_ClusterTendency + 3.343 × 10^−2^ × original_shape_Elongation + 1.608 × 10^−2^ × wavelet-LHL_glszm_ZoneEntropy–1.007 × 10^−1^ × log-sigma-5 mm-3D_glszm_ZonePercentage–2.254 × 10^−1^ × wavelet-LLH_firstorder_Skewness.

In total, 1037 radiomic features were extracted from the three-dimensional region of interest of the RCN lesion in the T2-weighted MRI sequences (Table S3). To address feature redundancy and identify the most predictive features, the Least Absolute Shrinkage and Selection Operator (LASSO) Cox regression algorithm was applied to the training cohort, which resulted in the selection of five features with nonzero coefficients (Figure 2). The radiomic signature was constructed by linearly combining these selected features, weighted by their respective coefficients from the LASSO model. An individualized radiomic score was computed for each patient based on this signature. The detailed calculation formula is provided in Methods S4.4 and Figure 2C.

The radiomic scores (median [interquartile range], Mann–Whitney U test) were significantly higher in RCN patients who developed herniation compared to those who did not, in both the training cohort (−0.53 [−2.44, 0.66] vs. 2.38 [0.55, 3.81], *p* < 0.001) and testing cohort (−0.65 [−1.89, 0.98] vs. 2.20 [0.86, 3.52], *p* = 0.004) (Figure S5). Univariate Cox regression analysis confirmed a significant association between the radiomic score and the risk of cerebral herniation (HR [95% confidence interval (CI)]: 1.49 [1.28, 1.73], *p* < 0.001). The robustness of this association was further supported by subgroup analyses stratified by sex, age, and RPE, which demonstrated consistent results across all subpopulations (Figure S6).

**Figure 3:**
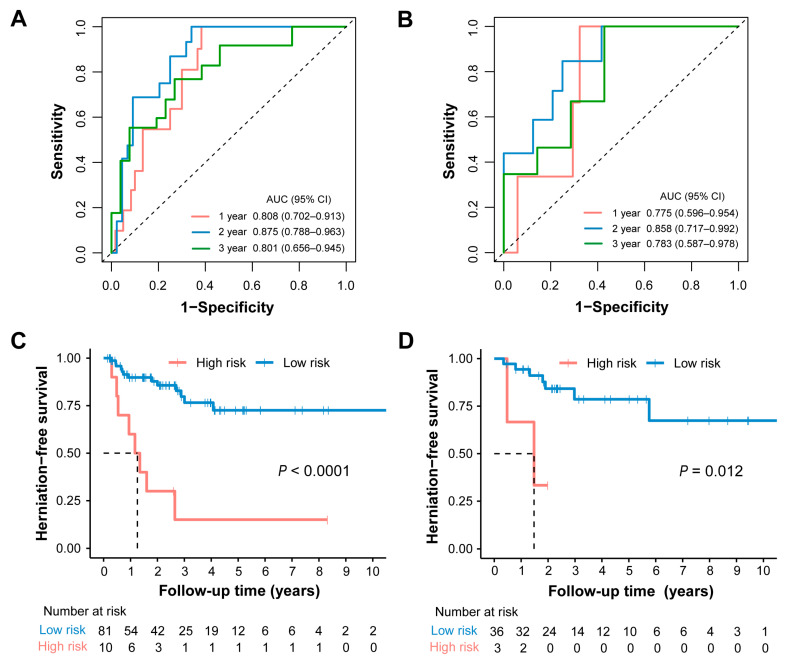
Performance assessment of radiomic signature. (**A**,**B**) The time–ROC curves showed that the radiomic signature achieved satisfactory prediction performances in the training (**A**) and testing (**B**) cohorts. (**C**,**D**) Survival curves according to radiomic signature in training (**C**) and testing (**D**) cohorts.

The predictive performance of the radiomic signature for cerebral herniation was evaluated using time-ROC analysis. In the training cohort, the area under the curve (AUC) for 1-, 2-, and 3-year herniation risk was 0.808 (95% CI: 0.702–0.913), 0.875 (95% CI: 0.788–0.963), and 0.801 (95% CI: 0.656–0.963), respectively (Figure 3A). The radiomic signature demonstrated comparable discriminative ability in the testing cohort, with corresponding AUCs of 0.775 (95% CI: 0.596–0.954), 0.858 (95% CI: 0.717–0.992), and 0.783 (95% CI: 0.587–0.978) for 1-, 2-, and 3-year risk, respectively (Figure 3B).

The optimal cutoff value of the radiomic score was determined to be 3.57 using the X-tile tool within the training set, stratifying patients into high- and low-risk groups. Subsequent Kaplan–Meier analysis revealed a significantly shorter herniation-free survival in the high-risk group compared to the low-risk group, a finding that was consistent in both the training (Figure 3C) and testing (Figure 3D) cohorts. These results further validate the strong discriminatory power of the radiomic signature for prognostic risk stratification.

### 3.3. Construction of the Radiomic Model

In the training cohort, univariate Cox regression analysis identified eight clinical variables and the radiomic score as being significantly associated with cerebral herniation. (Table S4). The significant clinical variables were: psychiatric symptoms, neutrophils, extensive perilesional edema, RPE, FLAIR hyperintensity, hemorrhage inside the cyst, necrosis mass, and history of corticosteroid therapy. Notably, baseline corticosteroid treatment at RCN diagnosis showed no significant association with herniation risk (HR [95% CI]: 1.776 [0.734, 4.296], *p* = 0.202), whereas a history of corticosteroid therapy prior to baseline was a significant predictor (HR [95% CI]: 2.415 [1.020, 5.713], *p* = 0.045).

Following multivariate Cox regression analysis with a backward stepwise selection procedure, a predictive model incorporating the radiomic score and RPE was established (Table 2). The variance inflation factor for both variables was 1.057, indicating the absence of substantial multicollinearity.

### 3.4. Model Evaluation

**Figure 4:**
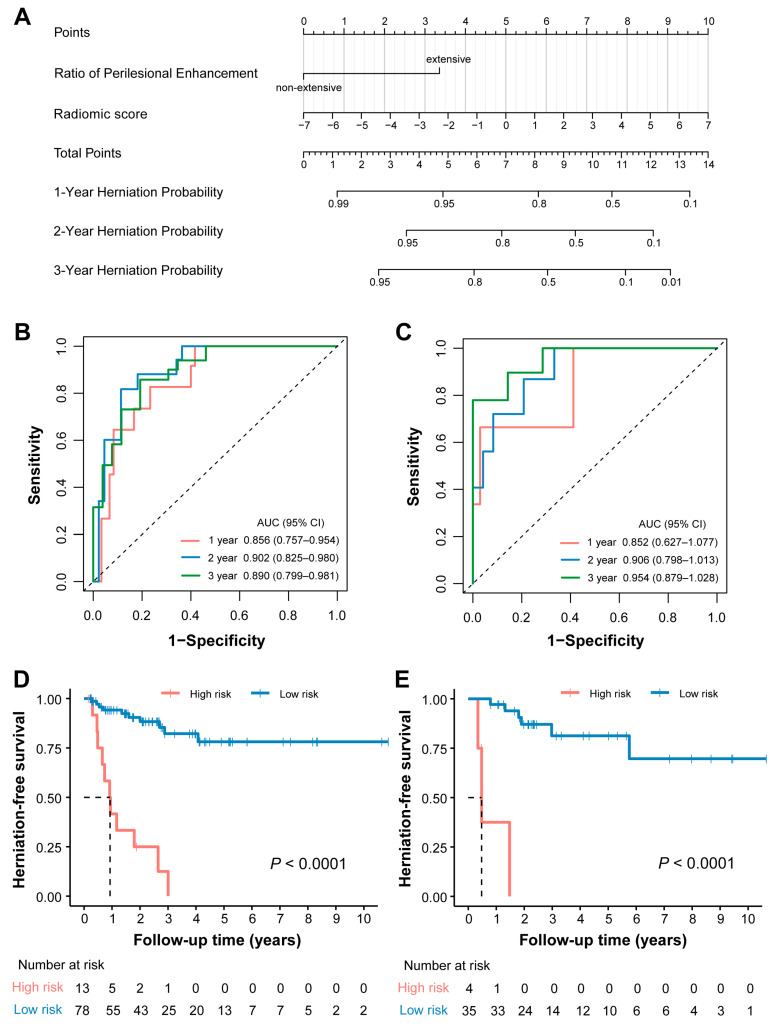
Development of nomogram and performance of radiomic model. (**A**) Radiomic nomogram developed to predict 1-, 2- and 3-year herniation-free survival for RCN. (**B**,**C**) The time–ROC curves showed that the radiomic model achieved satisfactory prediction performances in training (**B**) and testing (**C**) cohorts. (**D**,**E**) Survival curves according to model in training (**D**) and testing (**E**) cohorts.

**Figure 5:**
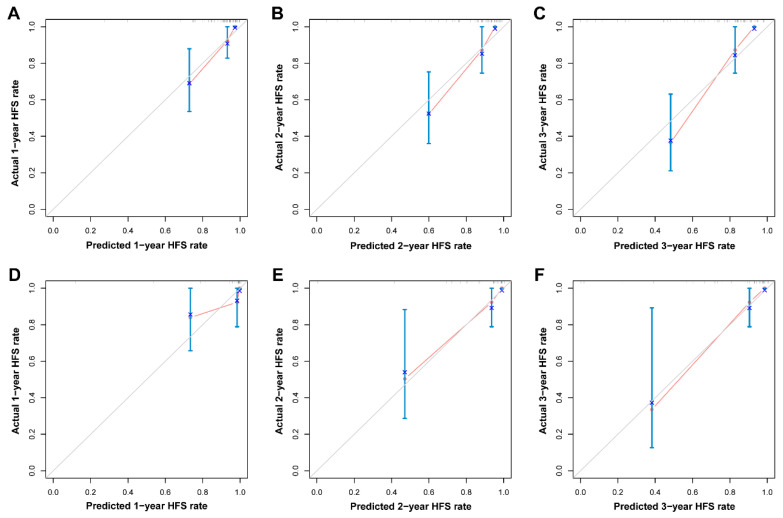
Calibration curves of the radiomic model. Calibration curves depict the calibration of the radiomic model in terms of consistency between the predicted and observed probability of HFS at 1-, 2- and 3-year in training (**A**–**C**) and testing cohorts (**D**–**F**). Abbreviations: HFS, herniation-free survival.

**Figure 6:**
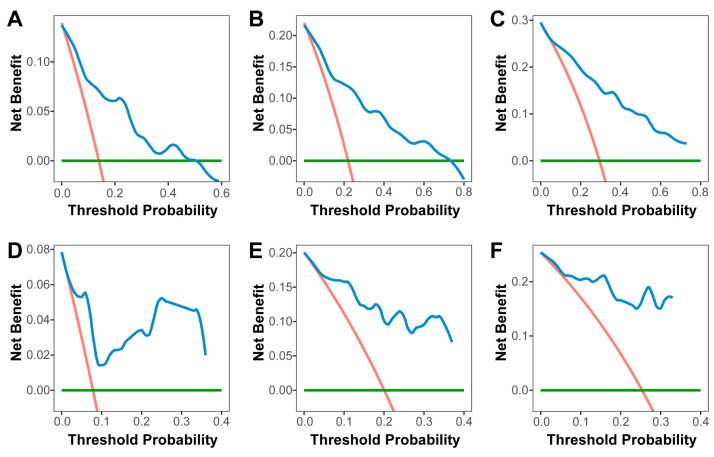
Decision curves of the model. Decision curves for herniation-free survival at 1 year (**A**), 2 years (**B**) and 3 years (**C**) in the training cohort, at 1 year (**D**), 2 year (**E**) and 3 years (**F**) in the testing cohort were applied to the model. The green lines depict the net benefit of a strategy of treating no patient. The red lines depict the net benefit of a strategy of treating all patients. The blue lines represent the net benefit of model-guided clinical decision-making, with closer to the top right of the figure indicating a greater benefit.

A radiomics-based nomogram was developed to facilitate individualized prediction of cerebral herniation risk at 1, 2, and 3 years (Figure 4A). The radiomic model demonstrated excellent discriminative ability, with a C-index of 0.841 (95% CI: 0.771–0.910) in the training cohort and 0.867 (95% CI: 0.776–0.958) in the validation cohort. Furthermore, the model exhibited superior predictive performance compared to the radiomic signature alone, as evidenced by time–ROC analysis. In the training cohort, the AUC values for predicting 1-, 2-, and 3-year herniation risk were 0.856 (95% CI: 0.757–0.954), 0.902 (95% CI: 0.825–0.980), and 0.890 (95% CI: 0.799–0.981), respectively (Figure 4B). Consistent performance was observed in the testing cohort, with corresponding AUC values of 0.852 (95% CI: 0.627–1.077), 0.906 (95% CI: 0.799–1.013), and 0.954 (95% CI: 0.879–1.029) at 1, 2, and 3 years, respectively (Figure 4C). Based on the final model, an individualized risk score was calculated for each patient using the following formula: Risk score = (Radiomic score × 0.318) + (RPE × 1.495). Applying an optimal cutoff value of 1.60, determined by X-tile analysis in the training cohort, patients were stratified into high- and low-risk groups. Among patients who developed brain herniation, the median follow-up time was 0.823 years (IQR: 0.479–1.400) in the high-risk group and 1.24 years (IQR: 0.568–1.970) in the low-risk group. Kaplan–Meier analysis confirmed that high-risk patients had significantly shorter herniation-free survival in both the training (log-rank *p* < 0.001; Figure 4D) and testing (log-rank *p* = 0.002; Figure 4E) cohorts. The calibration curves demonstrated satisfactory agreement between the predicted and observed probabilities of herniation at 1, 2, and 3 years in both the training and testing cohorts (Figure 5). Decision curve analysis further indicated that the use of the radiomic model for herniation prediction provided a superior net benefit compared to the treat-all or treat-none strategies across a wide range of threshold probabilities (Figure 6). The clinical applicability of the model is illustrated in Figure S7, which presents two representative cases where the model’s predictions were consistent with the actual clinical outcomes.

## 4. Discussion

As the most severe complication of radiation-induced brain injury, RCN may progress rapidly to cerebral herniation, posing a significant threat to patients’ survival. In this study, we developed a noninvasive, MRI-based radiomic model that integrates radiomic features and clinical variables to predict the risk of cerebral herniation in patients with established RCN. This model allows early identification of high-risk individuals, provides valuable prognostic information, and may support the development of personalized treatment strategies.

Radiomics enables the extraction of high-dimensional quantitative features from medical images to develop imaging biomarkers for diagnosis, prognosis, and clinical decision-support [22]. In the context of RCN, the lesions typically exhibit well-defined borders on T2WI, allowing for accurate delineation. Moreover, these lesions demonstrate considerable heterogeneity in internal signal intensity, spatial distribution, and morphological characteristics, which may reflect underlying pathophysiological processes driving disease progression. Based on these observations, we extracted a total of 1037 radiomic features from each lesion on T2WI. To manage the high-dimensional feature space, LASSO Cox regression was applied to identify five robust features and construct a radiomic signature for predicting cerebral herniation. Notably, this signature effectively stratified patients into high- and low-risk groups in both the training and testing cohorts.

To enhance predictive performance, we integrated clinical variables with the radiomic signature to develop a holistic model. We first investigated clinical factors associated with herniation risk. Although intravenous corticosteroids have long been used as a primary treatment for RIBI due to their anti-inflammatory and cytokine-modulating effects [23,24,25], our Cox regression analysis showed that corticosteroid therapy did not significantly reduce the risk of herniation. This aligns with previous studies suggesting that corticosteroids may not alter the underlying pathological progression of radiation-induced necrosis and offer limited clinical benefit [13]. Interestingly, however, patients who had received corticosteroid therapy prior to cyst formation showed a significantly different herniation risk compared to those who had not, indicating that corticosteroid-unresponsive RIBI may represent a more aggressive phenotype with a greater tendency toward herniation.

RPE, defined by enhancement of the cyst wall and adjacent lesions, was used to quantify the relative degree of perilesional enhancement. RPE was significantly associated with herniation in both univariable and multivariable analyses, suggesting its pathophysiological relevance. Following radiation injury, elevated VEGF expression promotes abnormal neovascularization characterized by disorganized, fragile, and hyperpermeable vessels [26]. Concurrently, radiation-triggered sterile inflammation and vascular injury contribute to blood–brain barrier disruption [27,28]. These processes collectively manifest as perilesional enhancement on imaging, with enhancement degree reflecting the severity of tissue injury and barrier breakdown [29,30]. Previous studies indicated that enhancing lesions are more likely to progress to necrosis, and cystic lesions often evolve from necrotic areas [6]. We thus hypothesized that marked perilesional enhancement reflects an “active” state in which the lesion periphery undergoes inflammation, tissue disintegration, necrosis, and liquefaction, promoting cystic expansion into adjacent brain parenchyma and increasing the risk of herniation.

To enhance the predictive power of the decision-support model, the radiomic signature was integrated with clinical characteristics [31]. Using a backward stepwise multivariate Cox regression approach, an MRI-based radiomic model was developed, incorporating two key predictors: the radiomic score and RPE. The model demonstrated satisfactory discriminative ability in both the training cohort (C-index: 0.841) and the testing cohort (C-index: 0.867). Patients were successfully stratified into high- and low-risk groups, with the high-risk group showing a significantly higher probability of cerebral herniation. Additionally, the model exhibited good calibration and provided considerable net benefit across a range of threshold probabilities. Collectively, these results suggest that our model may serve as a precise and reliable predictive tool for managing patients with RCN. Specifically, considering the short interval to herniation observed in the high-risk group (IQR: 0.479–1.400 years), we recommend a more intensive 3-month-interval imaging surveillance for these patients, while a 6-month follow-up interval may be sufficient for those identified as low-risk. Furthermore, we propose that early surgical resection of RCN could be a safe and beneficial strategy for high-risk patients to prevent potential herniation, yet it warrants further prospective studies for clinical validation. To our knowledge, this is the first study to develop a radiomic model for predicting herniation in nasopharyngeal carcinoma patients with RCN. Our study has several strengths. First, radiomic features were extracted from three-dimensional volumes of interest rather than two-dimensional regions, which enables better performance in capturing the heterogeneity of the entire lesion. Second, the integration of clinical variables with high-dimensional radiomic features yielded a model with superior predictive performance. Finally, the systematic investigation of clinical factors associated with cerebral herniation provides new insights into the mechanisms of disease progression.

Several limitations should also be acknowledged. First, its single-center retrospective design may introduce selection bias and limit the generalizability of the findings. Future validation using multi-center prospective datasets with larger sample sizes is warranted. Second, our model was developed for nasopharyngeal carcinoma patients with RCN. The generalizability to a broader spectrum of diseases, such as brain metastatic tumors treated with brain radiotherapy, requires future validation. Third, the exclusion of patients without follow-up cranial MRI at our institution may have led to an overestimation of herniation-free survival. Fourth, as cystic lesions progress dynamically, variations in RCN stages at baseline could introduce heterogeneity. Nevertheless, the model maintained robust predictive performance under real-world clinical conditions. Fifth, the reliance solely on T2-weighted imaging limits the capture of complementary pathophysiological information. Future studies could benefit from incorporating multi-parametric MRI data to further enhance predictive accuracy. Finally, the optimal follow-up intervals and surgical timing for patients at high risk of brain herniation warrant further prospective studies.

## 5. Conclusions

In conclusion, we developed and validated an MRI-based radiomic model that integrated radiomic signatures and clinical variables to predict the risk of cerebral herniation in patients with RCN following radiotherapy for nasopharyngeal carcinoma. The model demonstrated favorable discrimination, calibration, and clinical utility, suggesting its potential as a practical tool for individualized risk stratification and clinical decision-making. Nonetheless, further multi-center prospective studies are warranted to validate the performance of the model.

## Figures and Tables

**Table 1:** Characteristics of the patients in the training and testing cohorts.

Variable	Training Cohort (*n* = 91)	Testing Cohort (*n* = 39)	*p*-Value
Sex (male/female)	72/19	27/12	0.225
Age (years)	52.5 (8.8)	50.4 (9.5)	0.231
BMI (kg/m^2^)	22.1 (3.5)	21.3 (3.3)	0.241
Headache	33 (36.3%)	20 (51.3%)	0.110
Dizziness	35 (38.5%)	15 (38.5%)	>0.999
Dysarthria	15 (16.5%)	3 (7.7%)	0.184
Difficulty swallowing and coughing	37 (40.7%)	11 (28.2%)	0.178
Blurred vision	29 (31.9%)	10 (25.6%)	0.478
Diplopia	15 (16.5%)	3 (7.7%)	0.184
Memory decline	32 (35.2%)	8 (20.5%)	0.097
Personality change	9 (9.9%)	7 (17.9%)	0.245
Psychiatric symptoms	8 (8.8%)	5 (12.8%)	0.529
Epilepsy	33 (36.3%)	13 (33.3%)	0.749
SBP (mmHg)	124 (109.5, 134.5)	128 (115, 138)	0.334
DBP (mmHg)	79.6 (11.2)	79.6 (12.6)	0.984
Heart rate (bpm)	79.8 (12.9)	79.2 (11.8)	0.809
Hemoglobin (g/L)	129.8 (16.8)	127.6 (15.8)	0.484
Neutrophils (×10^9^/L)	3.8 (2.9, 5.3)	3.7 (2.9, 5.6)	0.799
Lymphocyte (×10^9^/L)	1.3 (1.0, 1.6)	1.2 (1, 1.7)	0.869
ALT (U/L)	19 (12, 25)	15 (11.5, 22.5)	0.191
Total cholesterol (µmol/L)	5.1 (4.4, 6.1)	5.4 (4.7, 6.2)	0.145
Triglyceride (µmol/L)	1.1 (0.8, 1.8)	1.1 (0.7, 1.8)	0.749
HDL (µmol/L)	1.2 (1, 1.5)	1.3 (1.1, 1.4)	0.913
LDL (µmol/L)	3.2 (2.5, 3.7)	3.4 (2.8, 4.2)	0.095
Hs-CRP (mg/L)	4.1 (1.5, 21.8)	3.6 (1.5, 12.4)	0.633
ESR (mm/h)	25 (12, 59)	17 (10, 39.5)	0.216
MoCA	22 (17, 26)	25 (17.5, 27.5)	0.216
LENT/SOMA	9 (3.5, 18)	12 (3.5, 22)	0.603
Cyst volume (cm^3^)	8.4 (3.1, 19.8)	7.5 (3.6, 13.2)	0.454
Total brain lesion volume (cm^3^)	26.3 (10.3, 50.1)	28.3 (9.9, 48.5)	0.875
Extensive perilesional edema	41 (45.1%)	21 (53.8%)	0.358
Communication with lateral ventricle	19 (20.9%)	5 (12.8%)	0.278
Ratio of perilesional enhancement			0.389
Extensive enhancement	71 (78.0%)	33 (84.6%)	
Non-extensive enhancement	20 (22.0%)	6 (15.4%)	
FLAIR signal intensity			0.619
Markedly hypointense	25 (27.5%)	12 (30.8%)	
Hypointense	45 (49.5%)	21 (53.8%)	
Isointense	15 (16.5%)	3 (7.7%)	
Hyperintense	6 (6.6%)	3 (7.7%)	
Hemorrhage inside the cyst	10 (11.0%)	4 (10.3%)	1.000
Necrosis mass	12 (13.2%)	5 (12.8%)	0.955
Cyst locularity			0.756
Unilocular	54 (59.3%)	22 (56.4%)	
Multilocular	37 (40.7%)	17 (43.6%)	
Nasopharyngeal carcinoma stage			0.367
Stage I–II	21 (23.1%)	5 (12.8%)	
Stage III	40 (44.0%)	21 (53.8%)	
Stage IV	30 (33.0%)	13 (33.3%)	
Tumor radiation dose (Gy)	70 (60, 72)	68 (60, 73)	0.700
Neck radiation dose (Gy)	61.2 (54, 66)	60 (50, 66)	0.661
Radiation approach			0.668
Conventional radiotherapy	55 (60.4%)	22 (56.4%)	
IMRT	36 (39.6%)	17 (43.6%)	
Received chemotherapy	53 (58.2%)	28 (71.8%)	0.144
Interval between radiotherapy and RIBI (years)	5 (3.2, 9.0)	6.5 (3.6, 9.9)	0.477
Interval between radiotherapy and RCN (years)	9.2 (7.2, 12.5)	9 (7.8, 11.2)	0.673
Corticosteroid treatment at baseline	46 (50.5%)	15 (38.5%)	0.206
History of corticosteroid therapy	29 (31.9%)	12 (30.8%)	0.902
Follow-up time (years)	1.9 (0.8, 3.8)	2.2 (1.6, 4.7)	0.063
Cerebral herniation	21 (23.1%)	9 (23.1%)	1.000
Radiomic score	−0.1 (2.7)	0.1 (2.9)	0.749

Note: The data are shown as the number (percentage), median (interquartile range) or mean (standard deviation). Abbreviations: BMI: body mass index; SBP: systolic blood pressure; DBP: diastolic blood pressure; ALT: alanine transaminase; HDL: high-density lipoprotein cholesterol; LDL: low-density lipoprotein cholesterol; Hs-CRP: high-sensitivity C-reactive protein levels; ESR: erythrocyte sedimentation rate; LENT/SOMA: Late Effects of Normal Tissue Subjective, Objective, Management; MoCA: Montreal Cognitive Assessment; IMRT: intensity-modulated radiation therapy; RIBI: radiation-induced brain injury; RCN: radiation-induced cystic brain necrosis.

**Table 2:** Univariate and multivariate Cox analyses of risk factors for cerebral herniation.

Variable	Univariable Cox Regression		Multivariable Cox Regression	
	Hazard Ratio (95% CI)	*p* Value	Hazard Ratio (95% CI)	*p* Value
Radiomic score	1.482 (1.242–1.768)	<0.001	1.374 (1.165–1.622)	<0.001
Psychiatric symptoms	4.153 (1.371–2.583)	0.012		
Neutrophils (×10^9^/L)	1.140 (1.017–1.277)	0.024		
Extensive perilesional edema	4.298 (1.653–11.174)	0.003		
RPE (extensive vs. non-extensive)	6.041 (2.515–14.510)	<0.001	4.460 (1.785–11.143)	0.001
FLAIR hyperintensity	5.013 (1.614–15.567)	0.005		
Hemorrhage inside the cyst	5.282 (2.019–13.821)	0.001		
Necrosis mass	5.672 (2.346–13.709)	<0.001		
History of corticosteroid therapy	2.415 (1.020–5.713)	0.045		

Abbreviations: CI: confidence interval, RPE: ratio of perilesional enhancement, FLAIR: Fluid-Attenuated Inversion Recovery.

## Data Availability

Data are available from the corresponding author upon reasonable request.

## Supplementary Materials

The following supporting information can be downloaded at https://www.mdpi.com/article/10.3390/cancers18060953/s1: Methods S1: Patient inclusion and exclusion criteria; Methods S2: Variables collected at baseline; Methods S3: MRI diagnostic criteria of herniation secondary to cystic brain necrosis [32,33,34,35,36,37]; Methods S4: Workflow [38,39,40]; Table S1: Definitions of imaging features of RCN at baseline; Table S2: Packages used for data analysis; Table S3: Extracted radiomic features; Table S4: Unadjusted Cox regression model of time to hernia in training cohort; Figure S1: Study flowchart of cohort selection; Figure S2: MRI demonstrate typical appearances of RCN; Figure S3: Imaging examples of RCN-induced cerebral herniation; Figure S4: Representative MRI demonstrating different RPE; Figure S5: Boxplots of the radiomic score; Figure S6: Subgroup analyses of the association between radiomic scores and cerebral herniation; Figure S7: Two representative cases to show radiomic model as a tool to predict outcomes of RCN.
