# Supplementary material for: A Novel Radiomic Model for Risk Stratification of Cerebral Herniation in Radiation-Induced Cystic Brain Necrosis
Source: Cancers (Basel). 2026 Mar 14;18(6):953. doi: 10.3390/cancers18060953 (PMC13024666; doi:10.3390/cancers18060953)
Supplement: Supplementary file 1 [file cancers-18-00953-s001.zip › cancers-4173031-supplementary.pdf]

Supplementary Materials

Methods S1. Patient inclusion and exclusion criteria. 2
Methods S2. Variables collected at baseline. 2
Methods S3. MRI diagnostic criteria of herniation secondary to cystic brain necrosis. 2
Methods S4. Workflow. 2
Methods S4.1. Imaging acquisition, preprocessing and segmentation. 2
Methods S4.2. Extraction of radiomic features 3
Methods S4.3. Radiomic feature extraction reproducibility 3
Methods S4.4. Construction of a radiomic signature and assessment of performance 3
Methods S4.5. Construction of the Radiomic model 4
Methods S4.6. Model evaluation 4
Table S1. Definitions of imaging features of RCN at baseline 6
Table S2. Packages used for data analysis 7
Table S3. Extracted radiomic features. 8
Table S4. Unadjusted Cox regression model of time to cerebral herniation in training cohort. 10
Figure S1. Study flowchart of cohort selection. 12
Figure S2. MRI demonstrate typical appearances of RCN 13
Figure S3. Imaging examples of RCN-induced cerebral herniation. 14
Figure S4. Representative MRI demonstrating different RPE 15
Figure S5. Boxplots of the radiomic score. 16
Figure S6. Subgroup analyses of the association between radiomic scores and cerebral herniation. 17
Figure S7. Two representative cases to show radiomic model as a tool to predict outcomes of RCN. 18

Supplemental Methods.

Methods S1. Patient inclusion and exclusion criteria.

Patients were eligible for inclusion if they met the following criteria: a) aged ≥18 years and completed radiotherapy (±chemotherapy) for histologically confirmed nasopharyngeal carcinoma, b) cranial MRI demonstrates a well-formed RCN, which was required to have a clearly defined boundary and a volume greater than 1cm³.

Patients were excluded based on the following criteria: a) history of surgical brain lesion resection prior to enrollment, b) radiographic evidence of tumor recurrence or metastases, c) cystic lesions potentially caused by other neurological diseases, such as brain abscess or intracranial tumors, d) cerebral herniation caused by RIBI at baseline, e) immediate surgical resection at baseline, f) absence of follow-up cranial MRI performed at least 3 months after baseline.

Methods S2. Variables collected at baseline.

The demographic, clinical, biochemical and imaging data were collected at baseline. Detailed baseline information was extracted from electronic medical records, including demographic data (date of birth, sex, BMI); medical history (neurological symptoms, co-existing illnesses, and brain surgery); vital signs; laboratory tests (hemoglobin, neutrophils, lymphocyte, alanine transaminase (ALT), serum lipids, high-sensitivity C-reactive protein levels (Hs-CRP), erythrocyte sedimentation rate (ESR); neuropsychological scale (Montreal Cognitive Assessment score (Moca), the Late Effects of Normal Tissue/Subjective, Objective, Management, Analytic scale score (LENT/SOMA)); cranial MRI assessments (Table S3); prior tumor-related information (nasopharyngeal carcinoma stage according to the seventh edition of the AJCC/UICC staging system, the commencement of radiotherapy (RT), RT dose and techniques, chemotherapy); diagnosis date of RIBI; diagnosis date of RCN; treatment details (history of corticosteroids therapy for RIBI before baseline, corticosteroids therapy at baseline) were also collected. Medical records reviewers were not aware of patient outcomes.

Methods S3. MRI diagnostic criteria of herniation secondary to cystic brain necrosis.

Cerebral herniation secondary to RCN can be divided into subfalcine hernia and uncal herniation (descending transtentorial hernia) on the basis of the location, which is diagnosed by professional radiologist: (A) The subfalcine hernia was defined as shift of midline structures: the distance from the midline to the septum pellucidum ≥ 5mm at the level of the septum pellucidum, which was relative to survival in many kinds of neurological disases.[1–5] (B) Uncal herniation was defined as meeting both of the following criteria: 1) compression (effacement) of the suprasellar cistern, 2) displacement, rotation, and elongation of brainstem.[1,6] Figure S3 showed several examples of RCN causing herniation.

Methods S4. Workflow.

Methods S4.1. Imaging acquisition, preprocessing and segmentation.

All enrolled patients in Sun Yat-sen Memorial Hospital underwent MRI using a 1.5T MR scanner (Gyroscan Intera; Philips, Aachen, Germany) with an 8-channel phased-array head coil at baseline and

follow-up. On T2-weighted cranial MRI, RCN lesion exhibits markedly hyperintense signal, with well-defined borders separating from radiation-induced brain edema and normal brain tissue, which allows the brain lesion margins to be delineated more accurately. Therefore, the baseline T2-weighted Digital Imaging and Communications in Medicine (DICOM) images were collected and used for analysis. The axial T2WI acquisition parameters were as follows: slice thickness, 5 mm; gap, 6 mm; echo time, 110 ms; repetition time, 4430 ms; and echo train length, 16 ms.

Before segmenting the radiomic features, the images were preprocessed, which consisted of three steps. First, intensity inhomogeneities in MRI were corrected by the N4 Bias Field Correction function. Secondly, all images were normalized by z-score transformation. Finally, we used linear interpolation to resample the images to 1 mm × 1 mm × 1 mm.

Volumes of interest (VOI) of the lesion were semi-automatically segmented using the *GrowCut* segmentation algorithms implemented in 3D Slicer software (version 5.6.2, http://www.slicer.org (accessed on 13 March 2026)). For patients with bilateral RCN lesions, the larger and better-defined lesion was selected for delineation. Then, radiologists meticulously edit the boundary of the ROI slice-by-slice manually, improving the alignment of the ROI with the lesion outlines. All segmentations were performed by a radiologist (Zhaoxi Cai) and revised by a senior radiologist (Mingwei Xie).

Methods S4.2. Extraction of radiomic features

The candidate radiomic features were extracted from each VOI using the open-source package PyRadiomics (version 3.1.0, https://pyradiomics.readthedocs.io/en/latest/ (accessed on 13 March 2026)), including first-order statistics features, shape- and size-based features, statistics-based textural features, features after wavelet transform, and LoG filtered features. Radiomic features in each class are described below and listed in Table S4. More detailed descriptions of each feature are available in the pyradiomics documentation at http://pyradiomics.readthedocs.io/en/latest (accessed on 13 March 2026). Target region intensity values were discretized using a bin width of 25.

In total, 1037 radiomic features were extracted from each lesion on T2-weighted images. Then, the z-score method was used to normalize the features and eliminate the difference in numerical scale for further analysis.

Methods S4.3. Radiomic feature extraction reproducibility

To evaluate the inter-observer reproducibility of radiomic features, we randomly selected 20 MRI images from the cohort. Two experienced neuroradiologists, Zhaoxi Cai and Mingwei Xie, segmented and extracted the radiomic features in a blinded fashion. Inter-observer reproducibility was quantified using the inter-class correlation coefficient (ICC), and ICC $\geq$ 0.80 was considered as substantial agreement and excellent reproducibility.[7] As a result, the mean ICC of our study was 0.924 (±SD, ±0.078), indicating favorable inter-observer feature extraction reproducibility.

Methods S4.4. Construction of a radiomic signature and assessment of performance

The Least Absolute Shrinkage and Selection Operator (LASSO) is a powerful method for regression with high dimensional predictors. In our study, the LASSO method was combined with Cox regression model for analysis of the risk of herniation, which could select the most important predictive features from the training set. LASSO is particularly suited for this study as it utilizes an L1 penalty to

perform simultaneous regularization and variable selection, effectively handling multicollinearity among features and preventing model overfitting.[8] The tuning parameter (λ) in LASSO model was determined via 10-fold cross-validation based on the minimum partial likelihood deviance, and the non-zero coefficients were defined as the weight of the selected feature, representing the correlation between the features and outcome. Finally, the radiomic signature was constructed to estimate the probability of cerebral herniation for each patient using the radiomic score, which was calculated by a linear combination of the selected features and coefficient vectors as follow:

$$Radiomic\ score\ = \sum_{i} Coefficient(feature_i) * Value(feature_i)$$

Radiomic scores were compared between patients with and without cerebral herniation using the Wilcoxon rank-sum test. Stratified analyses were also performed within different subgroups of all patients.

The timeROC analysis was performed to evaluate the predictive ability of radiomic scores for 1, 2, and 3 years. Patients were subsequently stratified into high- and low-risk groups according to the individual radiomic score, using the optimal cut-off value determined in the training cohort with X-tile software. Kaplan-Meier survival curves were generated for each risk group, and log-rank tests were applied separately in the training and testing cohorts to compare survival differences.

Methods S4.5. Construction of the Radiomic model

To construct the radiomic model, all clinical characteristics and radiomic signatures of each individual were examined initially using univariate Cox regression analysis in the training set. The significant variables with $P < 0.05$ were subsequently entered into multivariable Cox regression analysis. Feature selection for the final prediction model was conducted using a backward stepwise approach based on the Akaike Information Criterion[9]. A variance inflation factor was used to estimate the collinearity diagnostics. Finally, the radiomic model was built on the basis of independent risk factors of multivariate Cox regression analysis.

Methods S4.6. Model evaluation

The radiomic nomogram was generated to visualize the radiomic model and graphically evaluate variable importance. The concordance index (C-index), greater than 0.75 suggesting satisfactory discrimination, was calculated to assess the discrimination of the radiomic model. The timeROC analysis was used to assess the model’s predictive ability for 1, 2, and 3 years. Then, the risk score was calculated for each patient in the cohort based on the radiomic model. We categorized patients into high- or low-risk groups based on individual risk scores with an optimal cut-off in the training set using the X-tile software. We conducted log-rank tests separately in the training and testing cohort to compare the Kaplan-Meier curves of high and low-risk groups. In addition, the calibration curves (Hosmer-Lemeshow test) were performed to analyze the diagnostic performance of the radiomic model in both training and testing cohorts. Decision curve analysis was conducted to determine the clinical usefulness of the model by quantifying the net benefits at different threshold probabilities on the overall cohort.

References

1. Riveros Gilardi, B.; Muñoz López, J.I.; Hernández Villegas, A.C.; Garay Mora, J.A.; Rico Rodríguez, O.C.; Chávez Appendini, R.; De La Mora Malváez, M.; Higuera Calleja, J.A. Types of Cerebral Herniation and Their Imaging Features. *RadioGraphics* **2019**, *39*, 1598–1610, doi:10.1148/rg.2019190018.
2. Gao, G.; Wu, X.; Feng, J.; Hui, J.; Mao, Q.; Lecky, F.; Lingsma, H.; Maas, A.I.R.; Jiang, J. Clinical characteristics and outcomes in patients with traumatic brain injury in China: a prospective, multicentre, longitudinal, observational study. *Lancet Neurol.* **2020**, *19*, 670–677, doi:10.1016/S1474-4422(20)30182-4.
3. Coleman, M.E.; Roessler, M.E.H.; Peng, S.; Roth, A.R.; Risacher, S.L.; Saykin, A.J.; Apostolova, L.G.; Perry, B.L. Social Enrichment on the Job: Complex Work with People Improves Episodic Memory, Promotes Brain Reserve, and Reduces the Risk of Dementia. *Alzheimers Dement.* **2023**, alz.13035, doi:10.1002/alz.13035.
4. Jeon, S.-B.; Kwon, S.U.; Park, J.C.; Lee, D.H.; Yun, S.-C.; Kim, Y.-J.; Ahn, J.-S.; Kwun, B.-D.; Kang, D.-W.; Choi, H.A.; et al. Reduction of Midline Shift Following Decompressive Hemicraniectomy for Malignant Middle Cerebral Artery Infarction. *J. Stroke* **2016**, *18*, 328–336, doi:10.5853/jos.2016.00262.
5. Pullicino, P.M.; Alexandrov, A.V.; Shelton, J.A.; Alexandrova, N.A.; Smurawska, L.T.; Norris, J.W. Mass Effect and Death from Severe Acute Stroke. *Neurology* **1997**, *49*, 1090–1095, doi:10.1212/WNL.49.4.1090.
6. Ropper, A.H. Lateral displacement of the brain and level of consciousness in patients with an acute hemispheral mass. *N. Engl. J. Med.* **1986**, *314*, 953–958, doi:10.1056/NEJM198604103141504.
7. Landis, J.R.; Koch, G.G. The Measurement of Observer Agreement for Categorical Data. *Biometrics* **1977**, *33*, 159–174.
8. Tibshirani, R. The Lasso Method for Variable Selection in the Cox Model. *Stat. Med.* **1997**, *16*, 385–395, doi:10.1002/(sici)1097-0258(19970228)16:4<385::aid-sim380>3.0.co;2-3.
9. Collins, G.S.; Reitsma, J.B.; Altman, D.G.; Moons, K.G.M. Transparent Reporting of a Multivariable Prediction Model for Individual Prognosis or Diagnosis (TRIPOD): The TRIPOD Statement. *BMJ* **2015**, *350*, g7594, doi:10.1136/bmj.g7594.

Supplemental Tables.

Table S1. Definitions of imaging features of RCN at baseline.

Characteristic	Definitions
Cyst volume (cm^3)	Volume of the cystic lesion extracted from RCN radiomic features.
Total brain lesion volume (cm^3)	Volume of the total RIBI lesion extracted from RIBI radiomic features.
Perilesional enhancement volume (cm^3)	Volume of the enhancement surrounded the walls of RCN (segmented and quantified using 3D Slicer software).
Edema volume (cm^3)	Total brain lesion volume - Cyst volume.
Ratios of perilesional enhancement	Defined in **Materials and methods.**
Extensive perilesional edema	Defined as "present" if: 1) cyst volume < $5cm^3$ and the edema volume > $5cm^3$; or 2) cyst volume $\geq 5cm^3$ and the edema volume > the cyst volume; Otherwise defined as "absent".
Communicate with lateral ventricle	Whether the RCN communicates with the lateral ventricle.
FLAIR signal intensity	Signal intensity of the RCN on T2 FLAIR imaging. 1) hyperintense: higher than the surrounding edema lesion; 2) isointense: equal to the surrounding edema lesion; 3) hypointense: lower than surrounding edema lesion, while higher than CSF; 4) markedly hypointense: approximately equal to CSF.
FLAIR hyperintensity	Defined as "present" if the RCN showed a markedly hypointense signal (approximately equal to CSF) on FLAIR imaging; otherwise defined as "absent".
Hemorrhage inside the cyst	Presence of hemorrhagic deposition within the cystic lesion.
Necrosis mass	Presence of necrotic mass around the RCN.
Cyst locularity	Defined as "unilocular" if RCN appears as a single cystic cavity without internal septations; defined as "multilocular" if RCN contains internal septations or consists of multiple interconnected cystic cavities.

Abbreviations: RCN: radiation-induced cystic brain necrosis; RIBI: radiation-induced brain injury; FLAIR: Fluid-Attenuated Inversion Recovery; CSF: cerebrospinal fluid.

Table S2. Packages used for data analysis.

Application	R packages	Version
LASSO Cox regression	glmnet	4.1-8
Concordance index (C-index)	survcomp	1.56.0
Time-dependent ROC curves	timeROC	0.4
Variance inflation factor (VIF)	car	3.1-3
Kaplan-Meier curves	survival, survminer	3.6-4, 0.5.0
Nomogram construction	rms	8.0-0
Calibration plots	rms	8.0-0
Decision curve analysis (DCA)	dcurves	0.5.0

Table S3. Extracted radiomic features.

Group	Subgroup	Radiomic Features
First-order statistics features		Interquartile Range, Skewness, Uniformity, Median, Energy, Robust Mean Absolute Deviation, Mean Absolute Deviation, Total Energy, Maximum, Root Mean Squared, 90th Percentile, Minimum, Entropy, Range, Variance, 10th Percentile, Kurtosis, Mean
Shape- and size-based features		Elongation, Flatness, Least Axis Length, Major Axis Length, Maximum 2D Diameter Column, Maximum 2D Diameter Row, Maximum 2D Diameter Slice, Maximum3DDiameter, Mesh Volume, Minor Axis Length, Sphericity, Surface Area, Surface Volume Ratio, Voxel Volume
Statistics-based textural features	GLCM	Inverse Difference, Inverse Difference Normalized, Inverse Difference Moment, Inverse Difference Moment Normalized, Informal Measure of Correlation 1, Informal Measure of Correlation 2, Inverse Variance, Joint Energy, Joint Entropy, Joint Average, Maximal Correlation Coefficient, Maximum Probability, Sum Average, Sum Entropy, Sum Squares, Difference Entropy, Difference Variance, Difference Average, Autocorrelation, Cluster Shade, Cluster Prominence, Cluster Tendency, Contrast, Correlation,
	GLRLM	Short Run Low Gray Level Emphasis, Gray Level Variance, Low Gray Level Run Emphasis, Gray Level Non-Uniformity Normalized, Run Variance, Gray Level NonUniformity, Long Run Emphasis, Short Run High Gray Level Emphasis, Run Length NonUniformity, Short Run Emphasis, Long Run High Gray Level Emphasis, Run Percentage, Long Run Low Gray Level Emphasis, Run Entropy, High Gray Level Run Emphasis, Run Length Non-Uniformity Normalized
	GLSZM	Gray Level Variance, Zone Variance, Gray Level Non-Uniformity Normalized, Size Zone Non-Uniformity Normalized, Size Zone Non-Uniformity, Gray Level Non-Uniformity, Large Area Emphasis, Small Area High Gray Level Emphasis, Zone Percentage, Large Area Low Gray Level Emphasis, Large Area High Gray Level Emphasis, High Gray Level Zone Emphasis, Small Area Emphasis, Low Gray Level Zone Emphasis, Zone Entropy, Small Area Low Gray Level Emphasis
	NGTDM	Coarseness, Complexity, Strength, Contrast, Business
	GLDM	Gray Level Variance, High Gray Level Emphasis, Dependence Entropy, Dependence NonUniformity, Gray Level Non-Uniformity, Small Dependence Emphasis, Small Dependence High Gray Level Emphasis, Dependence Non-Uniformity Normalized, Large Dependence Emphasis, Large Dependence Low Gray Level Emphasis, Dependence Variance, Large Dependence High Gray Level Emphasis, Small Dependence Low Gray Level Emphasis, Low Gray Level Emphasis

Wavelet features[a]	wavelet(LLL)_x, wavelet(LLH)_x, wavelet(LHL)_x, wavelet(LHH)_x, wavelet(HLL)_x, wavelet(HLH)_x, wavelet(HHL)_x, wavelet(HHH)_x
LoG filtered features[a]	LoG(σ=3)_x, LoG(σ=5)_x

[a] denotes the first-order statistics features and statistics-based textural features listed above.

Abbreviations: LoG: Laplacian of Gaussian; GLSZM: Gray Level Size Zone Matrix; GLRLM: Gray Level Run Length Matrix; GLDM: Gray Level Dependence Matrix; GLCM: Gray Level Cooccurence Matrix; NGTDM, neighboring gray tone difference matrix.

Table S4. Unadjusted Cox regression model of time to cerebral herniation in training cohort.

Variable	Hazard Ratio (95%CI)	*P* Value
Sex (male vs. female)	0.779 (0.301–2.014)	0.606
Age (years)	0.994 (0.943–1.048)	0.833
BMI (kg/m^2)	1.059 (0.917–1.223)	0.436
Headache	1.748 (0.742–4.117)	0.201
Dizziness	0.935 (0.386–2.263)	0.881
Dysarthria	0.593 (0.23–1.532)	0.281
Difficulty swallowing and coughing	0.593 (0.230–1.532)	0.281
Blurred vision	2.332 (0.978–5.559)	0.056
Diplopia	1.450 (0.427–4.927)	0.551
Memory decline	2.133 (0.899–5.063)	0.086
Personality change	1.794 (0.524–6.139)	0.352
Psychiatric symptoms	4.153 (1.371–12.583)	0.012[a]
Epilepsy	0.727 (0.281–1.883)	0.511
SBP (mmHg)	1.006 (0.986–1.027)	0.57
DBP (mmHg)	1.021 (0.981–1.062)	0.314
Heart rate (bpm)	1.008 (0.972–1.045)	0.658
Hemoglobin (g/L)	1.003 (0.976–1.030)	0.845
Neutrophils (×10^9/L)	1.140 (1.017–1.277)	0.024[a]
Lymphocyte (×10^9/L)	0.924 (0.510–1.675)	0.795
ALT (U/L)	1.008 (0.975–1.043)	0.630
Total cholesterol (μmol/L)	1.114 (0.848–1.464)	0.438
Triglyceride (μmol/L)	0.781 (0.480–1.272)	0.321
HDL (μmol/L)	1.108 (0.777–1.581)	0.571
LDL (μmol/L)	1.299 (0.774–2.180)	0.321
Hs-CRP (mg/L)	1.000 (0.988–1.013)	0.944
ESR (mm/h)	0.991 (0.977–1.005)	0.219
Moca	1.029 (0.955–1.110)	0.452
LENT/SOMA	1.049 (1.006–1.094)	0.024
Cyst volume (cm3)	1.005 (0.978–1.034)	0.704
Total brain lesion volume (cm3)	1.001 (0.998–1.005)	0.383
Extensive perilesional edema	4.298 (1.653–11.174)	0.003[a]
Communicate with lateral ventricle	0.571 (0.168–1.938)	0.369
RPE (extensive vs. non-extensive)	6.041 (2.515–14.510)	<0.001[a]
FLAIR signal intensity		
markedly hypointense	Reference	
hypointense	1.700 (0.540–5.349)	0.364
isointense	1.004 (0.183–5.524)	0.996
hyperintense	6.906 (1.670–28.568)	0.008[a]

FLAIR hyperintensity	5.013 (1.614–15.567)	0.005[a]
Hemorrhage inside the cyst	5.282 (2.019–13.821)	0.001[a]
Necrosis mass	5.672 (2.346–13.709)	<0.001[a]
Cyst locularity (unilocular vs. multilocular)	0.912 (0.394–2.301)	0.150
Nasopharyngeal carcinoma stage		
Stage I-II	Reference	
Stage III	0.769 (0.262–2.257)	0.632
Stage IV	0.820 (0.250–2.692)	0.744
Tumor radiation dose (Gy)	0.996 (0.968–1.025)	0.795
Neck radiation dose (Gy)	1.006 (0.984–1.028)	0.608
Radiation approach (IMRT vs. conventional radiotherapy)	0.603 (0.221–1.650)	0.325
Received chemotherapy	0.733 (0.311–1.728)	0.478
Interval between radiotherapy and RIBI	0.960 (0.860–1.072)	0.472
Interval between radiotherapy and RCN	0.895 (0.786–1.019)	0.095[a]
Corticosteroids treatment at baseline	1.776 (0.734–4.296)	0.202
History of corticosteroids therapy	2.415 (1.020–5.713)	0.045[a]
Radiomic score	1.482 (1.242–1.768)	<0.001[a]

[a]$P < 0.05$.

Abbreviations: BMI: body mass index; SBP: systolic blood pressure; DBP: diastolic blood pressure; ALT: alanine transaminase; HDL: high-density lipoprotein cholesterol; LDL: low-density lipoprotein cholesterol; Hs-CRP: high-sensitivity C-reactive protein levels; ESR: erythrocyte sedimentation rate; LENT/SOMA: Late Effects of Normal Tissue Subjective, Objective, Management; MoCA: Montreal Cognitive Assessment; RPE: ratio of perilesional enhancement; IMRT: intensity modulated radiation therapy; RIBI: radiation-induced brain injury; RCN: radiation-induced cystic brain necrosis.

Supplemental Figures.

Figure S1. Study flowchart of cohort selection.

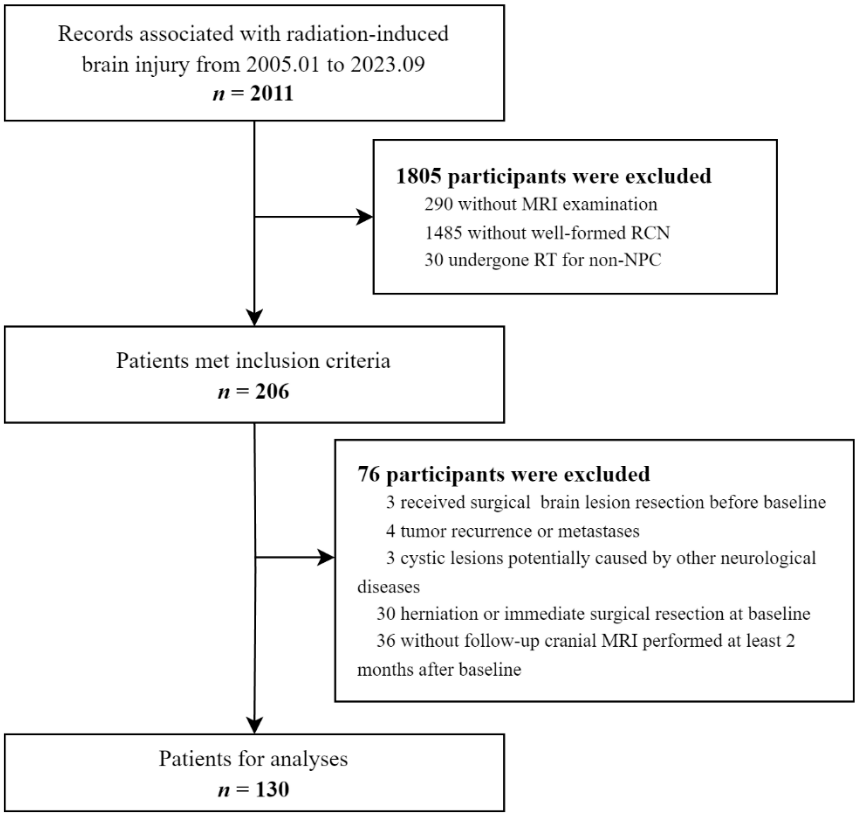

Abbreviations: RT: Radiotherapy; NPC: nasopharyngeal carcinoma; RCN: radiation-induced cystic brain necrosis.

Figure S2. MRI demonstrate typical appearances of RCN

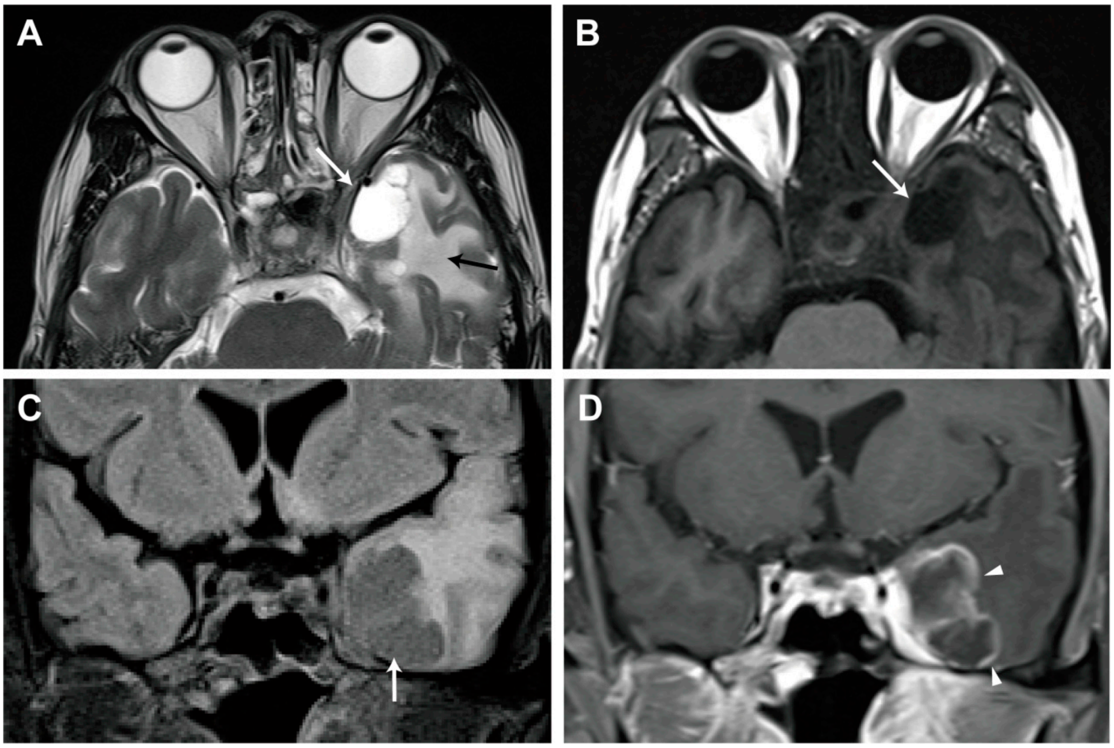

(A) Axial T2WI shows an oval, well-defined cyst with markedly hyperintense signal (white arrows), isointense to cerebrospinal fluid, surrounded by white matter lesions exhibiting mildly hyperintense signal (black arrows). **(B)** Axial T1WI reveals a cyst with markedly hypointense signal (white arrows). **(C)** Coronal T2-weighted FLAIR image demonstrates a cyst with hypointense signal (white arrows). **(D)** Coronal postcontrast T1WI shows a cyst with rim enhancement (arrow). In some cases, rim enhancement of the cyst wall is absent.

Figure S3. Imaging examples of RCN-induced cerebral herniation.

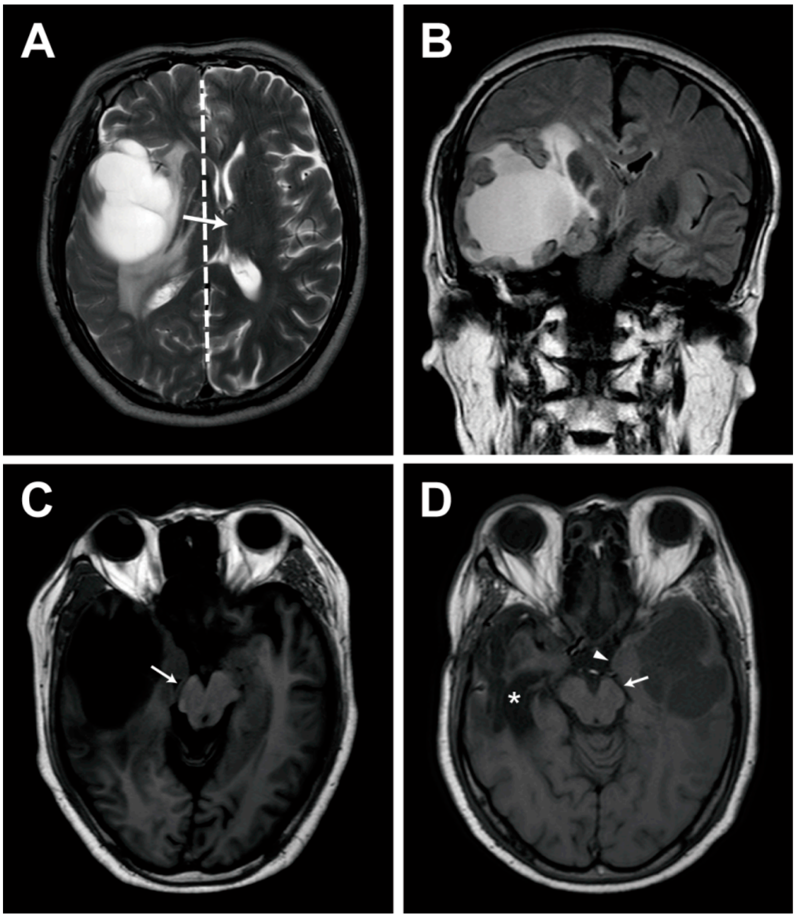

(A-B) RCN-induced subfalcine herniation. Axial T2WI (A) shows that the huge RCN displaces the septum pellucidum to the left (arrow) ≥ 5mm relative to the midline (dashed line). The same case is showed in the coronal T2-weighted FLAIR image (B). **(C-D) RCN-induced uncal herniation.** Axial T1WI shows compression of the suprasellar cistern (arrowheads), compression and rotation of the midbrain (straight arrow). Some shows the widening of the opposite ventricular atrium and temporal horn (*). Abbreviations: RCN, radiation-induced cystic brain necrosis.

Figure S4. Representative MRI demonstrating different RPE.

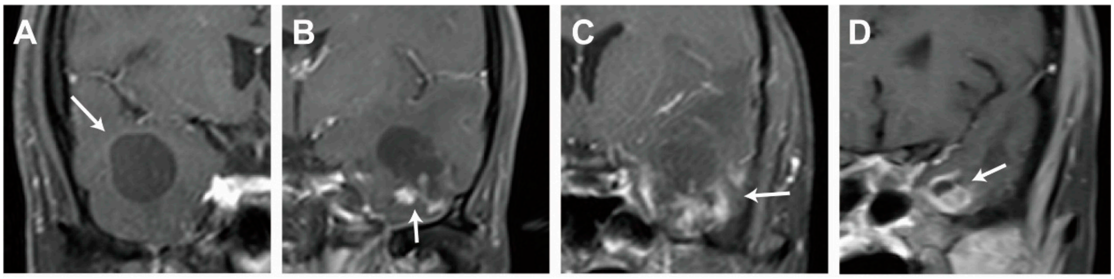

RPE reflects the extent of contrast enhancement surrounding the cystic lesion. **(A)** A cystic lesion without apparent perilesional enhancement (RPE: 0.02, non-extensive enhancement). **(B)** A cystic lesion with mild adjacent enhancement (RPE: 0.15, non-extensive enhancement). **(C)** A cystic lesion with substantial surrounding enhancement (RPE: 0.34, extensive enhancement). **(D)** A small cystic lesion with relatively prominent perilesional enhancement due to the small cyst volume (RPE: 0.51, extensive enhancement). Abbreviations: RPE, ratios of perilesional enhancement.

Figure S5. Boxplots of the radiomic score.

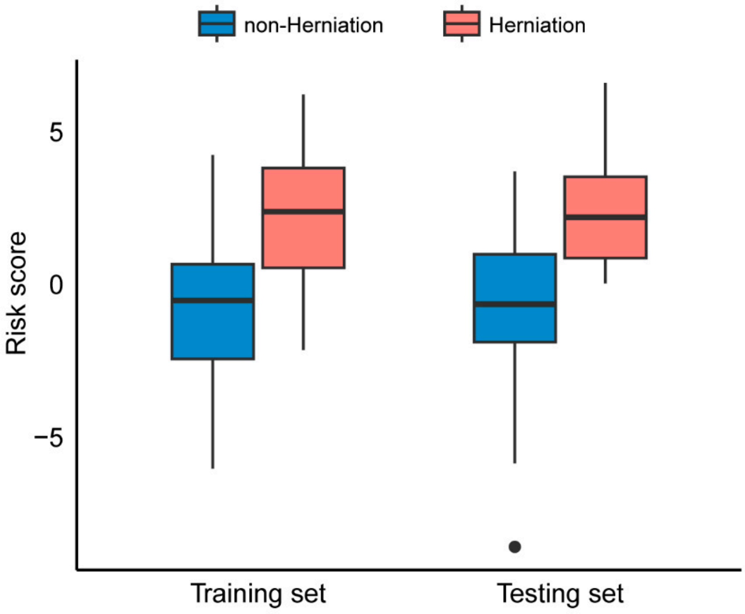

Based on the primary outcomes of follow-up, patients were stratified into herniation group and non-herniation group. The radiomic scores of herniation group were significantly higher than the non-herniation group both in the training cohort [-0.53 (-2.44, 0.66) vs. 2.38 (0.55, 3.81); $P < 0.001$] and the testing cohort [-0.65 (-1.89, 0.98) vs. 2.20 (0.86, 3.52); $P = 0.004$].

Figure S6. Subgroup analyses of the association between radiomic scores and cerebral herniation.

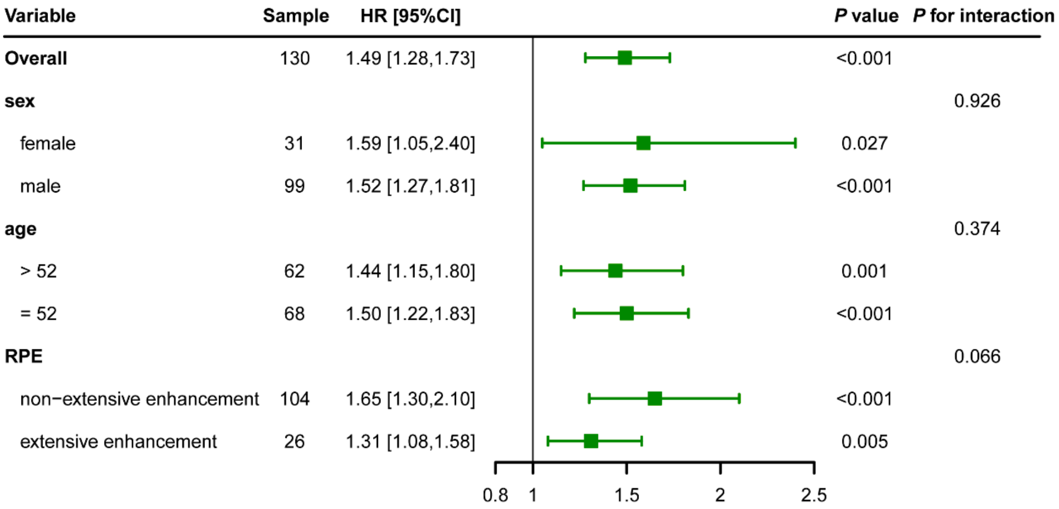

The forest plot shows the association between radiomic scores and cerebral herniation across different clinical subgroups, including sex, age, and RPE. The cutoff values of continuous variable (age) defining the subgroups were chosen by using the median values in all patients. Abbreviations: RPE, ratios of perilesional enhancement.

Figure S7. Two representative cases to show radiomic model as a tool to predict outcomes of RCN.

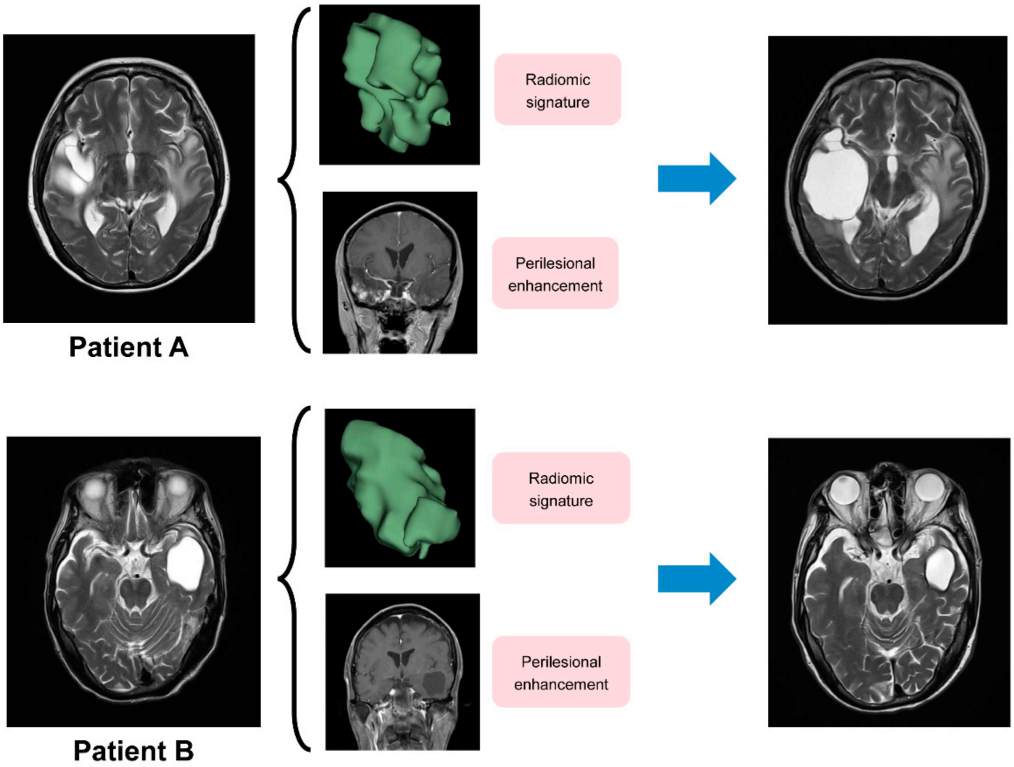

Patient A presented with a cystic lesion in the right temporal lobe at baseline. The radiomic signature derived from the lesion yielded a high radiomic score of 1.23 and marked perilesional ring enhancement was observed (RPE: 0.36). Based on the radiomic model, the patient was classified into the high-risk group. Follow-up MRI at 1.25 years revealed cerebral herniation with compression of the brainstem. **Patient B** presented with a cystic lesion in the left temporal lobe at baseline. The radiomic signature yielded a low radiomic score of 0.23 and no obvious perilesional enhancement was observed (RPE: 0.01). According to the radiomic model, the patient was classified into the low-risk group. During 4.2 years of follow-up, the cystic lesion remained stable and even decreased in size, without evidence of herniation. *Abbreviations*: RCN: radiation-induced cystic brain necrosis.
